# Phenotypic characterization of patients with ANCA-associated vasculitis and diffuse alveolar hemorrhage: a data-driven analysis of a multicentre, retrospective, nationwide cohort

**DOI:** 10.1016/j.aicoj.2026.100180

**Published:** 2026-10-05

**Authors:** Segolene Gendreau, Anne-Claire Sanna, Cyrielle Desnos, Romain Lombardi, Jolan Malherbe, Dimitri Titeca-Beauport, Florian Reizine, Raphael Porcher, Benjamin Terrier, Nicolas de Prost, on behalf of the PLEXHIA investigators

**Affiliations:** a Université Paris-Est-Créteil (UPEC), Faculté de Santé, Créteil, France; b Service de Médecine Intensive et Réanimation, Hôpitaux Universitaires Henri Mondor, DHU ATVB, 94000 Créteil, Assistance Publique - Hôpitaux de Paris, France; c Université Paris Est Créteil, Institut Mondor de Recherche Biomédicale, Groupe de Recherche Clinique CARMAS, 94000 Créteil, France; d Service de Médecine Interne, Hôpital Cochin, Assistance Publique - Hôpitaux de Paris, France; e Service de Médecine Intensive Réanimation, Hôpital Tenon, APHP Sorbonne Université, France; f Service de Médecine Intensive Réanimation, CHU de Nice, France; g Service de Médecine Intensive Réanimation, CHU de Caen Normandie, France; h Service de Néphrologie et Service de Médecine Intensive et Réanimation, CHU Amiens Picardie, France; i Service de Réanimation, Centre Hospitalier de Vannes, France; j Centre d’Épidémiologie Clinique, Hôtel-Dieu, AP-HP, Paris, France; k Université Paris Cité, F-75006 Paris, France; l INSERM U955, Team « Viruses, Hepatology, Cancer », Créteil, France

**Keywords:** ANCA-associated vasculitis, Alveolar hemorrhage, Intensive care

## Abstract

**Background:**

Severe diffuse alveolar hemorrhage (DAH) is a potentially fatal complication of ANCA-associated vasculitides (AAV). This study aimed to identify distinct clinical phenotypes among patients to the ICU, and to examine their association with mortality.

**Methods:**

We conducted an ancillary analysis of the national multicenter retrospective PLEXHIA cohort, including 197 patients with severe AAV-related DAH, defined by respiratory symptoms with new infiltrates consistent with alveolar hemorrhage, along with oxygen saturation ≤85%, high-flow oxygen or mechanical ventilation requirement. Kohonen self-organizing maps were applied to predefined clinical variables at ICU admission, excluding treatment and outcomes. Clusters were interpreted and compared regarding organ involvement, severity, management, and mortality at 30 and 90 days.

**Results:**

Between 2012 and 2024, 197 patients (66 years old, 52% female) were included, revealing marked clinical heterogeneity. Three phenotypes emerged: The MPO pulmonary-renal (MPO-PR) cluster included patients with anti-MPO ANCA, renal involvement and high oxygen requirements. The mild-respiratory (Mild-R) cluster included predominantly female patients with less severe respiratory compromise and mild renal involvement. The PR3 multi-organ (PR3-MO) cluster involved younger patients with anti-PR3 ANCA, severe acute respiratory failure and multiorgan failure. Renal replacement therapy was most frequent in the MPO-PR group (73%), while invasive mechanical ventilation was most common in the MPO-PR and PR3-MO groups (71% and 68%). Thirty-day mortality differed significantly: 23% (MPO-PR), 4% (mild-R cluster), and 14% (PR3-MO cluster).

**Conclusion:**

This large ICU cohort highlights three clinically relevant phenotypes of AAV-related DAH, which could inform the development of phenotype-driven immunosuppressive strategies.

**Key findings**

*What is already known on this topic*: Diffuse alveolar hemorrhage during ANCA-associated vasculitis is associated with substantial mortality and admission to the intensive care unit, but optimal therapeutic management is still not well characterized.

*What this study adds*: ICU patients with severe diffuse alveolar haemorrhage related to ANCA-associated vasculitis presented with three distinct clinical phenotypes, varying in terms of organ involvement, severity and short-term mortality.

*How this study might affect research, practice or policy*: These findings highlight the potential value of phenotype-driven, personalized immunosuppressive strategies to improve outcomes in this high-risk population.

## Introduction

Antineutrophil cytoplasmic antibody (ANCA)-associated vasculitides (AAV) comprise two main entities defined by ANCA specificity and clinical features, characterized by high morbidity and mortality rates: granulomatosis with polyangiitis (GPA) and microscopic polyangiitis (MPA) [1]. Among the most severe clinical presentations, acute respiratory failure (ARF) due to diffuse alveolar hemorrhage (DAH) is associated with substantial mortality and admission to the intensive care unit (ICU) [2,3]. Severe DAH, defined by an oxygen saturation of 85% or less, or the need for supplemental oxygen (≥6 L/min) or mechanical ventilation support, remains poorly studied in clinical trials, representing only 9% of patients included in the most recent randomized controlled trial [4]. Its clinical presentation and optimal therapeutic management are still not well characterized.

The management of DAH in AAV patients is primarily based on high-dose glucocorticoids (GCs) and immunosuppressive agents such as cyclophosphamide and/or rituximab. Supportive care measures, such as oxygen therapy or mechanical ventilation, are also employed, depending on the severity of respiratory impairment. Plasma exchange (PLEX) has been investigated in various clinical settings, producing inconsistent results [4,5]. However, once widely used for severe renal disease and DAH, it remains debated for respiratory involvement in this context [6], although selected high-risk subgroups may still derive benefit [7,8]. The PLEXHIA study [9] recently investigated whether PLEX reduced early mortality in patients with severe AAV-related DAH admitted to the ICU, using a target trial emulation approach. The study found no significant difference in mortality, which is consistent with current recommendations against the routine use of PLEX in this setting [6]. Whether subgroups of patients with DAH might benefit from a targeted approach has not been explored. One potential approach to identifying such subgroups involves the identification of distinct clinical phenotypes. The classification based solely on ANCA specificity appears insufficient to fully capture the heterogeneity in disease presentation and outcomes, particularly among critically ill patients. In fact, recent data from a large cohort of 3,868 patients with AAV identified five distinct clusters, challenging the conventional binary ANCA-based classification [10]. The phenotypic variability of critically ill patients with AAV-related DAH has not been specifically explored.

The objectives of this ancillary analysis of the PLEXHIA cohort were: (1) to use an unsupervised clustering method to identify distinct clinical phenotypes of patients with severe AAV-related DAH; and (2) to compare the hospital outcomes of these phenotypes.

## Methods

### Patient selection

The methods used for the constitution of the primary cohort have been described in detail previously [9]. Briefly, patients with severe AAV-associated DAH were included in a national, multicenter, retrospective observational study. Data were retrospectively collected by ICU and non-ICU physicians from internal medicine, nephrology, pulmonology, and rheumatology departments across 42 French centers affiliated with either the French Vasculitis Study Group (FVSG) or the *Groupe de Recherche Respiratoire en Réanimation Onco-Hématologique* (GRRR-OH).

Inclusion criteria were as follows: patients aged over 15 years who experienced a severe episode of DAH associated with either newly diagnosed or relapsing AAV (GPA or MPA) between November 2012 and January 2024. DAH was defined by the presence of respiratory symptoms (such as cough, dyspnea, or sputum production) in combination with new pulmonary infiltrates consistent with alveolar hemorrhage, and at least one of the following: hemoptysis, bronchoscopic findings indicative of DAH (including hemorrhagic bronchoalveolar lavage or a Golde score >100), or unexplained anemia. Severe DAH was defined by an oxygen saturation ≤85%, or the requirement for high-flow oxygen (≥6 L/min) or mechanical ventilation [4]. Patients were excluded if an alternative diagnosis for the pulmonary infiltrates was identified — such as infection or cardiogenic pulmonary edema — or if a decision to withhold intensive care treatment had been made at ICU admission. Because the primary outcome of the original cohort was to study the effect of PLEX on AAV-related DAH, additional exclusion criteria included any condition that independently required PLEX.

### Data collection

Data were collected from a standardized case report form including medical features (age, gender, medical history, presentation of vasculitis at diagnosis and relapse), DAH presentation, treatments and ICU management as well as outcomes (duration of mechanical ventilation, ICU and hospital length of stay, 30-day and 90-day mortality), and severity indexes, including the Simplified acute physiology score (SAPS II) [11] and the Birmingham vasculitis score (BVAS 2003, summarizing the organs involved) [12]. The Number of organs involved was calculated considering pulmonary (for all patients), renal, constitutional, musculoskeletal, skin, ear-nose-throat, eye, gastro-intestinal, neurological and cardiovascular manifestations.

### Statistical analysis

Clustering was performed using Kohonen’s self-organizing maps (SOM) via the Numero R package [13]. SOM is an unsupervised technique that enables the identification of patient subgroups based on multivariate patterns by organizing individuals with similar characteristics into neighboring regions of a two-dimensional map [14]. It provides an intuitive and concrete visual representation of datasets without intrinsic clustered structure through an unsupervised approach. The model was trained using a predefined set of predictor variables (see Supplementary Table S1, and supplementary methods, online resource), which are considered to be associated with disease presentation or severity. These included age [15], sex [16], ANCA specificity [10], comorbidities, vasculitis manifestations, severity scores (SAPS II [11], BVAS [17] and FFS [18]), and severe organ failure, reflected by invasive mechanical ventilation within 24 h of admission, and vasopressor and ECMO support. Outcome variables (mortality, duration of mechanical ventilation, and length of stay) and vasculitis treatments were intentionally excluded from the clustering input to avoid influencing subgroup formation (unsupervised clustering on pre-selected variables). The SOM algorithm arranged individuals on the map based on similarity, positioning those with comparable profiles closer together and facilitating visual and statistical identification of subgroups. High-intensity color regions on the SOM indicate significant local variation in data distribution, unlikely to have occurred by chance. Missing data were handled natively by the SOM algorithm without imputation. Quality control of the SOM output included visual inspection of data point distribution using histograms and direct assessment of potential outlier clusters.

Clusters of patients with similar profiles were subsequently reviewed and interpreted by two investigators (SG, NP). SOM visualizations were generated and inter-cluster comparisons were performed using standard statistical methods. The clusters were then labeled according to their main characteristics, in line with previous ANCA clustering analyses [10]. Categorical variables were expressed as counts and percentages and compared using the chi-squared test or Fisher’s exact test, as appropriate. Continuous variables were reported as medians with interquartile ranges (IQR) and compared using either ANOVA or Kruskal–Wallis test, depending on data distribution.

Survival analyses were performed between clusters for mortality at 30 and 90 days using the Log-rank test and Kaplan–Meier curves. All statistical tests were two-sided. The analysis adhered to the STROBE reporting guidelines [19]. Data were pooled and analyzed using R software version 4.1.0 (The R Foundation for Statistical Computing, Vienna, Austria) [20]. This study was conducted in accordance with Good Clinical Practice and the principles outlined in the Declaration of Helsinki. It was approved by the Ethics Committee of the French Intensive Care Society, Paris. No external funding was received for this study.

## Results

### Patient presentation and outcome

A total of 197 patients (median age 66 years [IQR, 53–74], 52% female) were included in the primary analysis. ANCA specificity was MPO-ANCA in 104 patients (53%) and PR3-ANCA in 94 patients (47%) (one patient had both MPO- and PR3-ANCA specificity). At the time of the DAH, 22 patients (11%) had underlying chronic kidney disease and 9 (5%) were on long-term renal replacement therapy (Table 1).

**Table 1:** Baseline characteristics of the 197 patients with AAV-vasculitis and intra-alveolar hemorrhage according to unsupervised clustering.

	Missing values	Whole sample (N = 197)	Cluster I MPO-PR N = 78	Cluster II Mild-R N = 69	Cluster III PR3-MO N = 50	*P value*
Age	0	66 [53−74]	71 [61−77]	60 [46−70]	64.5 [51−71]	
Sex (female)	0	103 (52.3)	34 (43.6)	44 (63.8)	25 (50)	
Vasculitis type:						
GPA	0	99 (50.3)	18 (23.1)	37 (53.6)	44 (88)	**<0.0001**
MPA	0	98 (49.8)	60 (76.9)	32 (46.4)	6 (12)	**<0.0001**
ANCA + PR3	0	94 (47.7)	13 (16.7)	37 (53.6)	44 (88)	
ANCA + MPO	0	104 (52.8)	65 (83.3)	33 (47.8)	6 (12)	
Past medical history						
Chronic respiratory failure	0	3 (1.5)	0 (0)	0 (0)	3 (6)	
Home oxygen therapy	0	1 (0.5)	0 (0)	0 (0)	1 (2)	0.228
Chronic heart failure	0	4 (2.0)	1 (1.3)	2 (2.9)	1 (2)	
Chronic kidney failure	0	22 (11.2)	12 (15.4)	4 (5.8)	6 (12)	
Chronic RRT	0	9 (4.6)	5 (6.4)	2 (2.9)	2 (4)	0.581
Diabetes	0	23 (11.7)	10 (12.8)	6 (8.7)	7 (14)	0.620
Immunosuppression	0	26 (13.2)	15 (19.2)	5 (7.2)	6 (12)	0.097
Obesity	0	26 (13.2)	10 (12.8)	11 (15.9)	5 (10)	0.635

Results are N (%), or medians (interquartile range); ANCA: Anti Neutrophil Cytoplasmic Antigen; PR3: Proteinase 3; GPA: Granulomatosis with polyangiitis, MPA: Microscopic polyangiitis; **Bolded** p-values are significant at the p < 0.05 level.

Training variables are presented only descriptively as they were used to derive cluster.

Hemoptysis was present in 135 patients (69%), with a median hemoglobin level of 7.6 g/dL [IQR, 6.6–8.6]. Higher respiratory support defining severe DAH included invasive mechanical ventilation in 102/197 patients (52%), non-invasive ventilation (HFNO or NIV) in 62/197 patients (31%), oxygen therapy via a non-rebreather mask in 10/197 patients (5%), and supplemental oxygen without a mask in 23/197 patients (12%). Renal involvement was observed in 173 of 188 patients (92%), with 106 patients presenting with serum creatinine levels greater than 300 µmol/L. Systemic symptoms were reported by 161 of 196 patients (82%), and the median BVAS score was 21 (IQR, 18–26) (Table 2).

**Table 2:** Disease characteristics of the 197 patients with AAV-vasculitis and intra-alveolar hemorrhage according to unsupervised clustering.

	Missing values	Whole sample (N = 197)	Cluster I MPO-PR N = 78	Cluster II Mild-R N = 69	Cluster III PR3-MO N = 50	*P value*
Radiologic infiltrates	0	178 (90.4)	68 (87.2)	62 (89.9)	48 (96.0)	0.253
Golde score	148	131 [100−185]	170 [126−182]	131 [956−202]	100 [72−113]	0.180
Haemoptysis	0	135 (68.5)	51 (65.4)	43 (62.3)	41 (82)	
Haemoglobin at DAH diagnostic	17	7.6 [6.6−8.6]	7.9 [6.7−9.0]	7.3 [6.5−8.7]	7.7 [6.6−8.2]	0.440
Maximal FiO2 (%)	28	60 [40−75]	66 [48−70]	58.5 [39−70]	66 [50−90]	
O_2_ by nasal canula or face mask	0	16 (8.1)	4 (5.1)	12 (17.4)	0 (0)	**0.001**
Non rebreather mask	3	109 (56.2)	48 (61.5)	30 (44.8)	31 (63.3)	0.066
Chest CT pattern						
Pulmonary nodule	0	31 (15.7)	12 (15.4)	7 (10.1)	12 (24)	0.122
Excavated nodule	0	12 (6.1)	2 (2.6)	8 (11.6)	2 (4)	0.057
Condensations	0	110 (55.8)	42 (53.8)	36 (52.2)	32 (64)	0.396
Other vasculitis manifestations						
Kidney	9	173 (92.0)	73 (100)	57 (85.1)	43 (89.6)	
Highest creatinine level (µM)	16	330 [164−512]	442 [257−564]	253 [100−406]	260 [119−485]	
Highest creatinine >300 µM	7	106 (55.8)	54 (72)	31 (46.3)	21 (43.8)	**0.001**
Proteinuria	20	150 (84.8)	59 (88.1)	53 (81.5)	38 (84.4)	0.580
Haematuria	10	128 (68.5)	48 (65.8)	49 (73.1)	31 (66)	0.588
General signs	1	161 (82.1)	65 (84.4)	51 (73.9)	45 (90)	
Fever	1	86 (43.9)	36 (46.8)	23 (33.3)	27 (54)	0.065
Asthenia	1	141 (71.9)	55 (71.4)	46 (66.7)	40 (80)	0.277
Weight loss	1	61 (31.1)	26 (33.8)	16 (23.2)	19 (38)	0.185
Joint pain	2	50 (25.6)	9 (11.8)	18 (26.1)	23 (46)	**<0.0001**
Arthritis	1	10 (5.1)	2 (2.6)	3 (4.3)	5 (10)	0.169
Myalgia	1	27 (13.8)	9 (11.7)	9 (13)	9 (18)	0.587
Skin	0	29 (14.7)	10 (12.8)	5 (7.2)	14 (28)	
Purpura	0	23 (11.7)	7 (9)	5 (7.2)	11 (22)	**0.030**
ENT	1	46 (23.5)	6 (7.8)	9 (13)	31 (62)	
Chondritis	2	2 (1.0)	1 (1.3)	0 (0)	1 (2)	0.530
Nasal crusting	2	14 (7.2)	2 (2.6)	3 (4.3)	9 (18.4)	**0.002**
Eye	2	12 (6.2)	2 (2.6)	2 (2.9)	8 (16.3)	
Digestive tract	2	18 (9.2)	8 (10.4)	0 (0)	10 (20)	
Peripheral nerve	1	22 (11.2)	6 (7.9)	3 (4.4)	11 (22)	
Central nervous system	1	5 (2.6)	5 (6.5)	0 (0)	0 (0)	
Heart	0	16 (8.1)	12 (15.4)	2 (2.9)	2 (4)	
Organs involved*	13	4 [3,4]	4 [3,4]	3 [3,4]	5 [4,6]	**<0.001**
Five factor score	18	2 [2,3]	3 [2,3]	2 [2,2]	2 [1,2]	**<0.0001**
SAPS II score	27	42 [31−54]	49 [41−59]	31 [23−37]	43 [33−58]	
BVAS score 2003	30	21 [18−26]	22 [20−27]	20 [17–21]	26 [21−32]	

Results are N (%), or medians (interquartile range); FiO2: fraction of inspired oxygen; BAL: bronchoalveolar lavage; ENT: Ear-nose-throat; BVAS: Birmingham Vasculitis Activity Score; SAPS II: simplified acute physiology score; **Bolded** p-values are significant at the p < 0.05 level.

*Including pulmonary (all) and kidney, constitutional, musculoskeletal, skin, ear-nose-throat, eye, gastro-intestinal, neurological and cardiovascular manifestations.

Training variables are presented only descriptively as they were used to derive cluster.

At ICU admission, patients exhibited substantial disease severity, with a median SAPS II score of 42 (IQR, 31–54). Immunosuppressive treatment included PLEX in 154 patients (78%) and intravenous cyclophosphamide in 145 patients (74%, Supplementary Fig. S1). Within the first 24 h of ICU admission, 78 patients (40%) required invasive mechanical ventilation. The median duration of invasive and/or non-invasive ventilation was 9 days (IQR, 5–15). Neuromuscular blocking agents and prone positioning were used in 74 patients (38%) and 49/196 patients (25%). Vasopressor support was required in 79 patients (40%) and 76 out of 187 (41%) required renal replacement therapy for acute renal failure. The 30-day mortality rate was 14% (28 out of 197 patients) (Table 3). The SAPS II and Five Factor scores were higher in patients who had died by day 90 (Supplementary Table S2). The main causes of death were vasculitis flares within three months (12/40; 32%) and infections after three months (12/21; 52%).

**Table 3:** Treatment and ICU care of the 197 patients with AAV-vasculitis and intra-alveolar hemorrhage according to unsupervised clustering.

	Missing values	Whole sample (N = 197)	Cluster I MPO-PR N = 78	Cluster II Mild-R N = 69	Cluster III PR3-MO N = 50	*P value*
Vasculitis treatments						
PLEX	0	154 (78.2)	58 (74.4)	51 (73.9)	45 (90)	0.064
IV cyclophosphamide	0	145 (73.6)	58 (74.4)	50 (72.5)	37 (74)	0.964
Rituximab	0	65 (33)	22 (28.2)	24 (34.8)	19 (38)	0.478
Corticosteroid pulse	0	188 (95.4)	73 (93.6)	66 (95.7)	49 (98)	0.504
Organ support						
High flow oxygen	0	90 (45.7)	30 (38.5)	37 (53.6)	23 (46)	0.183
NIV	1	39 (19.9)	21 (27.3)	11 (15.9)	7 (14)	0.111
Invasive mechanical ventilation	0	102 (51.8)	55 (70.5)	13 (18.8)	34 (68)	**<0.0001**
IMV within 24 h	0	78 (39.6)	45 (57.7)	7 (10.1)	26 (52)	
MV duration (days)*	56	9 [5–15]	12 [6–16]	6 [3–9]	10 [6–18]	**0.001**
VAP	93	38 (36.5)	17 (30.4)	3 (21.4)	18 (52.9)	**0.044**
Neuromuscular blocking agents	0	74 (37.6)	41 (52.6)	8 (11.6)	25 (50)	**<0.0001**
Prone positioning	1	49 (25.0)	26 (33.3)	6 (8.8)	17 (34)	**0.001**
Inhaled nitric oxide	0	12 (6.1)	9 (11.5)	1 (1.4)	2 (4)	**0.030**
ECLS	0	16 (8.1)	3 (3.8)	0 (0)	13 (26)	
Catecholamines	0	79 (40.1)	46 (59)	4 (5.8)	29 (58)	
Norepinephrine	1	74 (37.8)	41 (53.2)	4 (5.8)	29 (58)	**<0.0001**
Epinephrine	1	3 (1.5)	0 (0)	0 (0)	3 (6)	**0.012**
Dobutamine	1	6 (3.1)	4 (5.2)	0 (0)	2 (4)	0.173
Acute RRT	10	76 (40.6)	41 (56.2)	15 (22.7)	20 (41.7)	**<0.0001**
RRT	1	102 (52.0)	57 (73.1)	17 (25)	28 (56)	**<0.0001**
Death by day 30	0	28 (14.2)	18 (23.1)	3 (4.3)	7 (14)	**0.005**
Death by day 90	0	40 (20.3)	25 (32.1)	7 (10.1)	8 (16)	**0.003**
ICU length of stay	33	13 [7–20]	13 [10–23]	7 [5–14]	13 [10−26]	**<0.0001**
Hospital length of stay	45	44 [34−56]	50 [35−65]	37 [28−49]	46 [39−59]	**0.001**

Results are N (%), or medians (interquartile range); PLEX: plasma exchange; IV: intravenous; NIV: non-invasive ventilation; IMV: invasive mechanical ventilation; VAP: ventilator acquired pneumonia; ECLS: extra-corporeal membrane oxygenation support; RRT: renal replacement therapy; ICU: intensive-care unit.

*Invasive or non-invasive. Bolded p-values are significant at the p < 0.05 level.

Training variables are presented only descriptively as they were used to derive cluster.

### Unsupervised clustering

Three distinct patient clusters were identified based on clinical characteristics on admission to the ICU using SOMs (Fig. 1; Table 1, Table 2, Table 3).

**Fig. 1:**
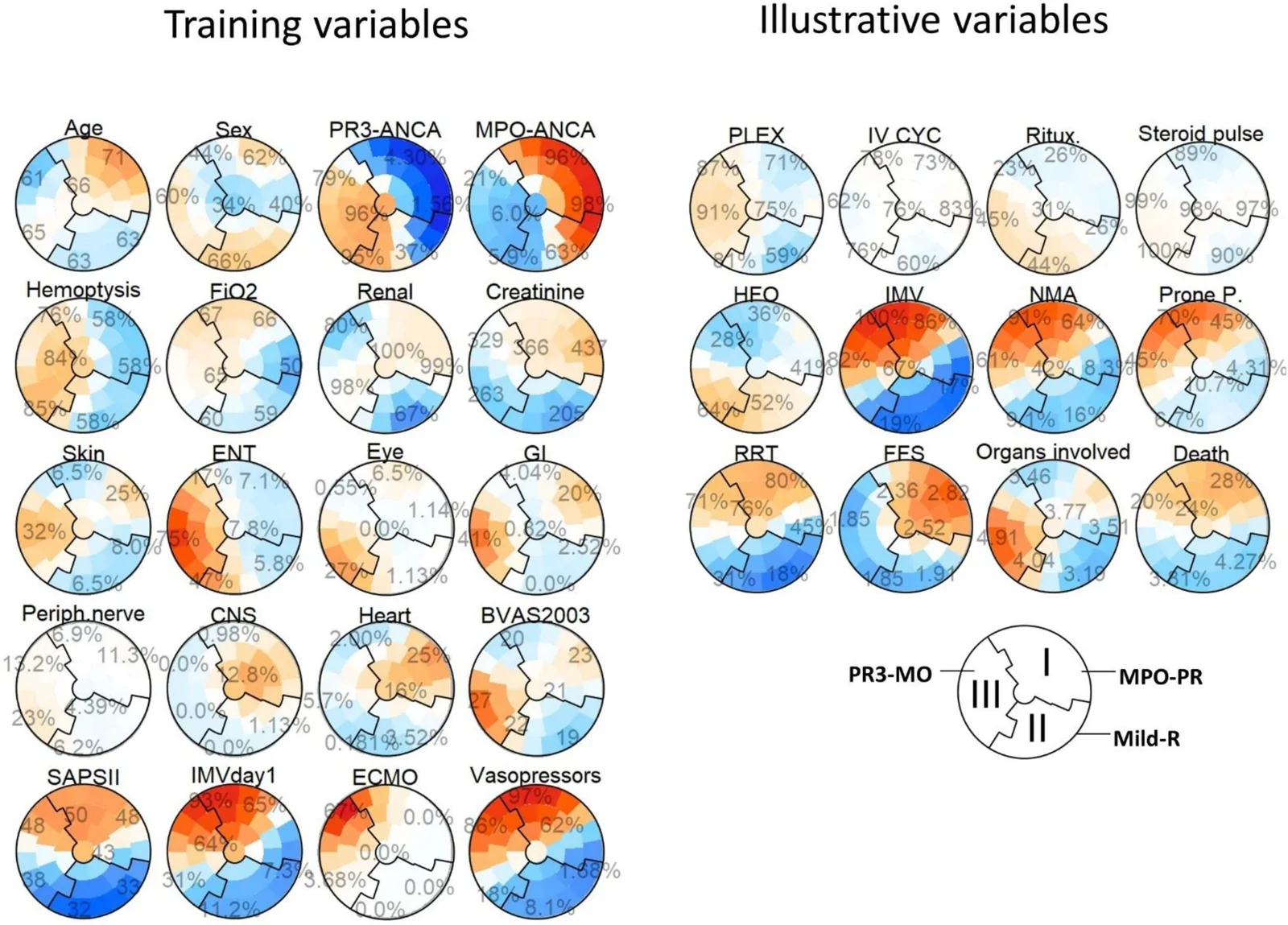
Self-organized maps of unsupervised analysis of the characteristics of 197 patients with AAV-vasculitis and intra-alveolar hemorrhage. I: Cluster 1, II: cluster 2; III: cluster 3; Patients with similar characteristics are located by the SOM algorithm closely on the maps, within 1 of the small districts. Each individual map shows the mean values or proportions per district for each characteristic: blue indicates the lowest average values, red the highest, with numbers shown for a selection of representative districts in each SOM. Unsupervised clustering divided the SOM in 3 clusters. Patients in cluster I for example are located in the upper right quadrant, and had older age, higher creatinine level, and more acute kidney injury. ANCA: Anti Neutrophil Cytoplasmic Antigen; PR3: Proteinase 3; GPA: Granulomatosis with polyangiitis, MPA: Microscopic polyangiitis; RRT: renal replacement therapy; GI: gastro-intestinal tract manifestations; CNS: central nervous system; PLEX: plasma exchange; IV CYC: intravenous cyclophosphamide; Ritux: rituximab; CTC: corticosteroids; HFO: high-flow oxygen; IMV: invasive mechanical ventilation; NMA: neuro-muscular blocking agent; Prone P: prone positionning; ECMO: extra-corporeal membrane oxygenation; BVAS: Birmingham Vasculitis Activity Score; SAPSII: simplified acute physiology score; FFS: five factors score. Organs involved included pulmonary (for all) and kidney, constitutional, musculoskeletal, skin, ear-nose-throat, eye, gastro-intestinal, neurological and cardiovascular

Cluster I included the oldest patients (median age: 71 years [IQR, 61–77]), with 15% (n = 12/78) having pre-existing chronic kidney disease, and a majority having anti-MPO ANCA. These patients had high oxygen requirements, 62% of them (n = 48/78) requiring a non-rebreather mask. All patients had renal involvement (100%, n = 73/73), with the highest recorded serum creatinine levels (median: 443 µmol/L [IQR, 257–564]). Half of the patients had fever, 10% (8/77) had gastro-intestinal involvement, and 12/78 (15%) had cardiac manifestations. The FFS and SAPS II scores were the highest in this cluster. Most of the patients received PLEX, while 71% (n = 55/78) required invasive mechanical ventilation. Fifty-nine percent of patients (n = 46/78) required vasopressor support, and 56% (n = 41/73) underwent acute renal replacement therapy (RRT). This cluster had the highest mortality rate, at 23% on day 30 (n = 18/78) and 32% at three months (n = 25/78). This group was named anti-MPO with pulmonary-renal syndrome (MPO-PR) cluster.

Patients in Cluster II were predominantly female (64%, n = 44/69) and had no chronic kidney disease or major comorbidities. They required less oxygen and although 85% of patients (n = 57/67) had renal involvement, creatinine levels were lower than in cluster I, with 46% of patients (n = 31/67) having creatinine values greater than 300 µmol/L. BVAS 2003 and SAPS II scores were the lowest of all clusters. PLEX was administered to 74% of patients (n = 51/69), and 19% of them (n = 13/69) required invasive mechanical ventilation. Vasopressor support was required in less than 6% of cases (n = 4/69), and this group had the lowest 30-day mortality at 4% (n = 3/69). Comparatively to other clusters of severe DAH requiring ICU admission, this group was named mild-respiratory (Mild-R) cluster.

Cluster III included younger patients (median age: 65 years [IQR, 51–71]). These patients had comorbidities such as diabetes (14%, n = 7/50) and chronic kidney failure (12%, n = 6/50). They mostly had anti-PR3 ANCA (88%, n = 44/50), and severe acute respiratory failure requiring invasive mechanical ventilation support in 34/50 (68%), with frequent use of neuromuscular blockade (50%, n = 25/50). Although FFS was low (2 [IQR: 1–2]), they commonly presented multi-visceral involvement, with a median of 5 [IQR: 4, 6] organs involved. They presented renal manifestations (90%, n = 43/48), although less than 50% (n = 21/48) had creatinine levels >300 µmol/L, skin (28%, n = 14/50), ENT (62%, n = 31/50), gastrointestinal (20%, n = 10/50), and peripheral nervous system (22%, n = 11/50) involvement. PLEX was administered to 90% of patients (n = 45/50), while 58% (n = 29/50) required vasopressor support and 26% (n = 13/50) underwent extra-corporeal membrane oxygenation. Despite this severity, only 42% of patients required acute RRT (n = 20/48). The 30-day mortality rate in this cluster was 14% (n = 7/50). This group was named anti-PR3 with multi-organ (PR3-MO) cluster.

No significant differences were observed between clusters regarding comorbidities other than chronic respiratory failure. However, the 30- and 90-day survival rates and survival probability over time differed significantly according to clusters (Table 3, Fig. 2, Supplementary Fig. S2), cluster I having the highest risk of death.

**Fig. 2:**
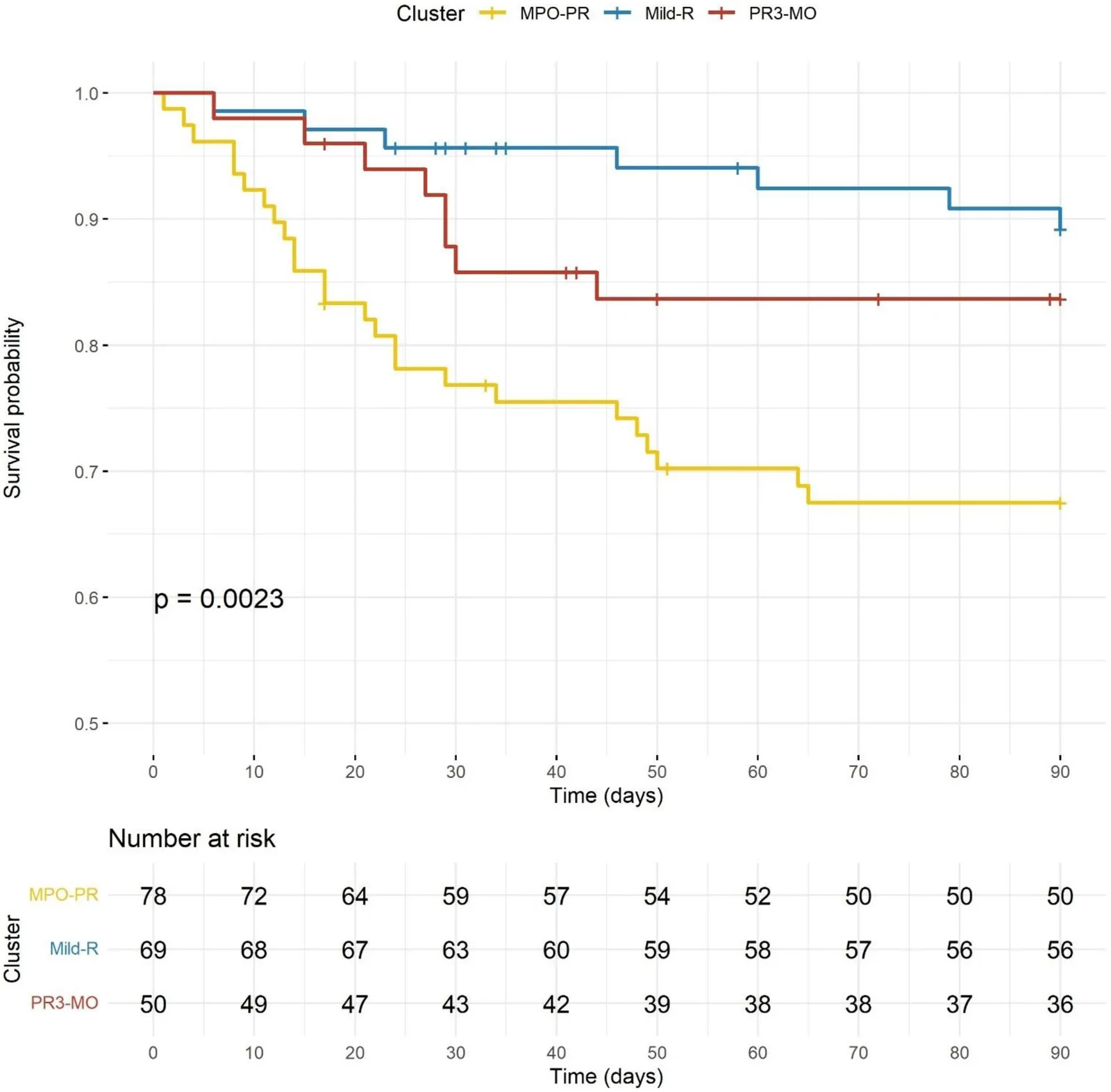
90-day Kaplan–Meier survival curve according to clustering of 197 patients with AAV-vasculitis and severe intra-alveolar hemorrhage.

## Discussion

This large cohort of patients with severe AAV related substantial heterogeneity, distributed into three distinct patient clusters with differing clinical presentations and outcomes: (i) elderly patients with anti-MPO ANCA and pneumo-renal syndromes, including severe acute kidney injury, often requiring renal replacement therapy, and with high mortality, (ii) non-comorbid patients with moderate oxygen requirements and renal insufficiency, mostly requiring continuous monitoring and with low mortality, and (iii) patients with anti-PR3 ANCA, and multivisceral involvement, including pneumo-renal syndromes with skin, ENT, gastro-intestinal or peripheral nervous system involvement.

To our knowledge, no clustering analysis of severe AAV with DAH has yet been reported. The SOM design enables the exploration of heterogeneity among these rare and difficult-to-study patients, identifying distinct presentation subgroups in an unsupervised manner. While preliminary and not sufficient to guide management, these data provide unique insights into the most severe cases. Gisslander et al. recently identified five prognostically relevant clusters in patients with AAV [10]. One of their renal-driven clusters (cluster SK), characterized by severe renal involvement, showed partial consistency with the MPO-PR cluster. Patients in this cluster had a mean age of 67 years and a median creatinine level of 579 µmol/L. Fifty-two percent of patients had respiratory involvement, 8% had cardiovascular involvement, and 31% died. Clusters with limited pulmonary involvement were excluded from the cohort by design. However, one of their clusters (IMS) shows some resemblance to our PR3-MO cluster, which is characterized by multisystemic involvement in young patients (mean age 55 years, 68% PR3-ANCA positive). Patients in this IMS cluster commonly exhibited pulmonary, peripheral nervous system, skin, and gastro-intestinal manifestations, and a mortality rate of 15% [10]. Identifying patient subgroups with similar profiles allows differentiation of individuals whose ICU trajectories vary markedly. Guiding future studies on optimal strategies within these clusters could help identify patients most likely to benefit from rescue therapies, such as plasma exchange, whose use remains debated [5,9].

A significant difference observed between the clusters was the sex ratio, with a higher proportion of females in cluster II. Lindberg et al. observed different clinical phenotypes in patients with AAV and classified patients according to GPA and MPA subtypes [21]. They observed a greater number of organ systems involved in GPA and highlighted sex differences in clinical presentation: among patients with GPA, males were more likely to have PR3-ANCA, as well as more frequent renal involvement, including end-stage renal disease, and pulmonary manifestations. In contrast, women with GPA were more likely to present with MPO-ANCA. No significant sex differences were found in patients with MPA, except for a later age at diagnosis in men [21]. Another important difference between the clusters was age. MPO-PR had the highest mortality rate and included the oldest patients. Advanced age is a well-established prognostic factor in critically ill patients in general and in AAV in particular, and is included in the revised Five-Factor Score for AAV [18]. In line with our findings, Kimmoun et al. reported a higher age in non-survivors compared to survivors [22]. Regarding vasculitis manifestations, although all patients in our cohort had respiratory involvement as an inclusion criterion, the severity of acute respiratory failure varied between clusters. MPO-PR and PR3-MO had the highest burden, with 71% and 68% of patients requiring invasive mechanical ventilation. These features are consistent with general ICU prognostic scores such as SAPS II [11], although they are not included in the revised FFS, unlike renal involvement, which was more pronounced in MPO-PR. Other distinguishing features of clustering included cardiac and gastrointestinal involvement, both components of the revised FFS [18], and nervous system manifestations. These patterns are consistent with the clustering of Lindberg et al. [21], who reported similar organ involvement in the general AAV population and within the GPA subgroup. In line with their findings, we observed a significant influence of ANCA serotype (PR3 vs. MPO) on clustering. PR3-ANCA was more frequently associated with multi-organ involvement and relapse, whereas MPO-ANCA was rather associated to renal-limited disease. However, no clear difference in patient survival has been observed between PR3-AAV and MPO-AAV [23]. The three identified clusters indeed did not map clearly onto either the traditional GPA versus MPA nor the PR3-ANCA versus MPO-ANCA classification. AAV is a highly heterogeneous disease, both in its clinical presentation and severity. The absence of a clear benefit of some therapeutic strategy across the disease as a whole [4] may mask a genuine benefit within specific clinical presentations [5], underscoring the importance of better characterizing these patient subgroups. These findings suggest finer granularity could be required. In line with the findings of Gisslander et al. who identified five distinct clusters [10], this study also identified a relatively milder cluster amongst severe DAH (the mild-R cluster), independent of ANCA specificity. This cluster could potentially benefit from less intensive therapeutic strategies. The combination of ANCA specificity, the pattern of organ involvement and severity captures a dimension of heterogeneity that neither classification system fully accounts for in isolation.

In our study, clustering was associated with mortality rates at 1 and 3 months. Kimmoun et al. reported a three-month mortality rate of 18%, comparable to that observed in our cohort, and particularly consistent with cluster III [22]. In their study, ICU mortality in patients with AAV was attributed to disease flare in 69% of patients and infection in 31%. It highlighted the double burden of immunosuppression-related complications, particularly in mechanically ventilated ICU patients, and uncontrolled disease activity. Finally, focusing specifically on DAH, Quartuccio et al. reported associated renal involvement in 73% of patients and an overall mortality of 18% after a median follow-up of 37 months [13–77] [3]. Prognostic factors in this study included age and need for respiratory support. Therefore, it remains an open question as to which cluster may benefit most from an intensified therapeutic approach. Indeed, this overview of the existing heterogeneity in this population, by offering a phenotypic characterization of patient’s heterogeneity, may help to better delineate the most severe phenotypes and inform discussions regarding therapeutic strategies and prognosis. Since more than 30% of day-90 deaths were attributable to vasculitis flare, further investigation is needed to determine which cluster would benefit from early ICU admission and more intensive immunosuppressive therapy.

This study has several limitations: first, as a post hoc analysis of a retrospective cohort, it may be subject to the inherent limitations of its retrospective design. The primary objective was not an in-depth analysis of baseline demographic factors. However, it is the largest reported cohort of severe DAH to date, with a low rate of missing data. Data extraction was performed locally by pairs of intensivists and specialists directly involved in the care of the patients, ensuring high data reliability and quality. Another limitation was the clustering process, which relied on a predefined set of variables, some of which had missing values. These variables were defined a priori by the authors according to their prognostic relevance, the rate of missing values and their clinical relevance. Although clustering was performed in an unsupervised manner, the reliance on pre-selected variables may nonetheless constitute a form of supervision in how the phenotypes were defined. In particular, because age, disease severity, and organ support are themselves associated with mortality, their inclusion may have contributed to defining clusters that partly reflect differences in overall severity. No patient was excluded from the SOM analysis due to a complete lack of training variables, allowing for the most robust stratification possible given the available data. The presence of outliers limited internal validation, suggesting that the three phenotypes were not sharply delineated and exhibited substantial overlap. It is consistent with the hypothesis that ANCA-associated vasculitis phenotypes lie along a continuum of disease severity and organ involvement rather than representing discrete disease categories, and the contribution of ANCA serotype to the observed clustering may have been overestimated. Although this process allowed the identification of clinically relevant subgroups, the external validity of these subgroups remains to be established. Moreover, excluding outcomes from the clustering analysis allowed us to construct phenotypes identifiable prospectively at the bedside, albeit at the expense of capturing phenotypes that might instead be defined by distinct trajectories of clinical evolution. Finally, the immunosuppressive regimen selection was comparable across subgroups, precluding any therapeutic conclusions. These findings open the discussion on whether a more aggressive therapeutic approach should be considered for the most severe forms (e.g., PR3-MO), while weighing the potential harm that such strategies may cause to the most fragile patients (e.g., MPO-PR). Prospective, phenotype-driven studies are warranted to assess whether tailored immunosuppressive strategies could improve outcomes across the identified clusters. Despite these limitations, this clustering-based stratification may support individualized therapeutic approaches in this rare and critically ill population where randomized controlled trials targeting specific subgroups are unlikely to be feasible.

## Conclusion

In this large multicenter cohort of critically ill patients with AAV-related DAH, unsupervised clustering revealed three candidate clinical phenotypes requiring external validation. The first phenotype comprised older patients with severe renal dysfunction and high mortality. The second phenotype included female patients with mild renal involvement. The third phenotype was constituted of younger patients with multiorgan failure. These findings underscore the importance of data-driven stratification in refining prognostic assessment and supporting tailored therapeutic strategies for this rare, high-risk population. While external validation is warranted, this approach could inform future research and improve clinical decision-making.

## Members of the PLEXHIA investigators group (to be included in the XML as collaborators)

C. Desnos, R. Lombardi, J. Malherbe, D. Titeca-Beauport, F. Reizine, G. Louis, K. Nassarmadji, J. Campagne, JP. Martellosio, A. Deroux, E. Bendiab, V. Coirier, J. Catano, M. Pineton De Chambrun, M. Samson, C. Vigneron, M. Samson, A. Neel, P. Smets, T. Deltombe, B. Brilland, M. Desgrouas, O. Simon, S. Faguer, S. Giorgiutti, R. Paule, F. Arrive, I. Masson, T. Quémeneur, CA. Durel, E. de Montmollin, JP. Quenot, F. Perrin, H. Lobbes, Q. Lajoye, R. Mesbah, R. Outh, B. Thoreau, A. Lacraz, C. Philipponnet, D. Contou, F. Parazols, F. Garo, G. Blonz, G. Ducoux, H. Yildiz, I. Rey, L. Di Ascia, N. Issa, P. Le Guen, V. Queyrel, R. Eid, R. Arcani, Y. Uzunhan, X. Puéchal.

## Author contributorship

ACS, SG, RP, NDP and BT prepared study protocol. ACS and SG extracted data, and SG and RP did the statistical analysis. ACS, SG, RP, NDP and BT wrote the manuscript. All authors had full access to all the data in the study and had final responsibility for the decision to submit for publication.

## Patient consent and ethical approval

This study was approved by the Ethics Committee of the French Intensive Care Society, Paris (IRB00014135, approval number: CE SRL 23-077).

## Funding

None.

## Data sharing statement

Anonymized data could be made available upon reasonable request to the corresponding author.

## Declaration of competing interest

Ségolène Gendreau reports support from Pharma Dom for attending a meeting, outside the submitted work; Nicolas de Prost has served as an advisor or speaker for Moderna and AstraZeneca; Benjamin Terrier reports consulting fees from CSL Vifor, AstraZeneca, GlaxoSmithKline, Novartis. Other authors have no conflicts of interest to disclose.

All authors have completed the ICMJE uniform disclosure form and declare: Ségolène Gendreau reports support from Pharma Dom for attending a meeting, outside the submitted work; Nicolas de Prost has served as an advisor or speaker for Moderna and AstraZeneca; Benjamin Terrier reports consulting fees from CSL Vifor, AstraZeneca, GlaxoSmithKline, Novartis.
