# Supplementary material for: Phenotypic characterization of patients with ANCA-associated vasculitis and diffuse alveolar hemorrhage: a data-driven analysis of a multicentre, retrospective, nationwide cohort
Source: Ann Intensive Care. 2026 Oct 5;16:100180. doi: 10.1016/j.aicoj.2026.100180 (PMC13677274; doi:10.1016/j.aicoj.2026.100180)
Supplement: Supplementary file 1 [file mmc1.docx]

Phenotypic characterization of patients with ANCA-associated vasculitis and diffuse alveolar hemorrhage: an unsupervised analysis of a multicentre, retrospective, nationwide cohort.

## Online Resource

S. Gendreau, MD^1,2,3*^, AC. Sanna, MD^4*^, B. Terrier†, MD, PhD^4^, N. de Prost†, MD, PhD^1,2,3,5^, on behalf of the French Vasculitis Study Group (FVSG) and the Groupe de Recherche en Réanimation Respiratoire du patient d'Onco-Hématologie (GrrrOH)

^1^Université Paris-Est-Créteil (UPEC), Créteil, France

^2^Service de Médecine intensive et réanimation, Hôpitaux Universitaires Henri Mondor, DHU ATVB, 94000 Créteil, Assistance Publique - Hôpitaux de Paris

^3^Université Paris Est Créteil, Faculté de Santé de Créteil, Institut Mondor de Recherche Biomédicale, Groupe de recherche clinique CARMAS, 94000 Créteil, France

^4^Service de Médecine Interne, Hôpital Cochin, Assistance Publique - Hôpitaux de Paris

^5^INSERM U955, Team « Viruses, Hepatology, Cancer », Créteil, France

### Supplementary Methods: SOM analysis

The SOM was constructed using the Numero R package version 1.9.0 ^1^. Training variables (n = 24; **Supplementary Table 1**) were preprocessed by centering and scaling continuous variables to unit variance and automatically detecting binary variables. Missing values were not imputed; the SOM algorithm accommodates missingness natively by computing inter-patient distances using only the variables available for each pair. All patients and all 24 candidate variables met the completeness criteria and were retained in the analysis.

The map was initialized by k-means clustering (187 subsamples per cycle, converging after 28 training cycles) and subsequently refined using the Kohonen self-organizing algorithm (187 subsamples per cycle, 67 training cycles until convergence). Map radius (r = 3) and smoothness (1.208) were set automatically according to the package's default. Distances between patients and map units were computed using Euclidean distance in standardized variable space.

Partitioning of the trained SOM into discrete subgroups was performed using the numero.subgroup function with its automatic segmentation mode, which applies a data-driven, algorithmic procedure based on statistically significant regional patterns identified via permutation testing (permutation-based significance, 16,496 permutations per variable, reference Z = 7.71) to partition map districts into subgroups without user input on boundary placement. This procedure selected a three-subgroup solution as the optimal partition for the dataset. No alternative cluster numbers were manually tested or selected by the investigators.

**References:**

1.	Kohonen T, Somervuo P. How to make large self-organizing maps for nonvectorial data. *Neural Netw* 2002;15(8–9):945–952.

### Supplementary Table 1: Training and illustrative variables for SOM algorithm

| **Training variables** | **Illustrative variables** |
| --- | --- |
| Age (years)  Sex (female)  ANCA+ PR3  ANCA+ MPO  Chronic respiratory failure  Chronic heart failure  Chronic kidney failure  Hemoptysis  Maximal FiO2 (%, 28 missing data)  Kidney manifestations (9 missing data)  Highest serum creatinine level (µM, 16 missing data)  General signs (1 missing data)  Skin manifestations  ENT manifestations (1 missing data)  Eye manifestations (2 missing data)  Digestive tract manifestations (2 missing data)  Peripheral nerve manifestations (1 missing data)  Central nervous system manifestations (1 missing data)  Heart manifestations  BVAS score 2003 (30 missing data)  SAPS II score (27 missing data)  IMV within 24 hours of ICU admission  ECLS  Catecholamines support | PLEX,  IV cyclophosphamide Rituximab  Corticosteroid pulse  High flow oxygen/ NIV  Invasive mechanical ventilation (IMV)  Neuromuscular blocking agents (NMA)  Prone positioning (PP)  Death by day 90  ICU length of stay  Hospital length of stay  Five factors score |

BVAS: Birmingham Vasculitis Activity Score; ECMO: extracorporeal membrane oxygenation support; ENT: ear – nose – throat; ICU: intensive care unit; IMV: invasive mechanical ventilation; SAPS II: Simplified Acute Physiology Score II;

### Supplementary Table 2: Demographics and cause of deaths of the 197 patients with AAV-vasculitis and intra-alveolar hemorrhage according to 90 days survival.

|  | **Day 90 survivors**  **(N=157)** | **Day 90 deceased**  **(N= 40)** | ***P value*** |
| --- | --- | --- | --- |
| Age | 65 [51, 72] | 68 [60, 77] | **0.02** |
| Sex (female) | 85 (54.1) | 18 (45.0) | 0.392 |
| GPA | 85 (54.1) | 14 (35.0) | **0.047** |
| MPA | 72 (45.9) | 26 (65.0) | **0.047** |
| ANCA+ PR3 | 79 (50.3) | 15 (37.5) | 0.203 |
| ANCA+ MPO | 79 (50.3) | 25 (62.5) | 0.23 |
| Chronic respiratory failure | 2 (1.3) | 1 (2.5) | > 0.99 |
| Chronic heart failure | 2 (1.3) | 2 (5.0) | 0.388 |
| Chronic kidney failure | 16 (10.2) | 6 (15.0) | 0.561 |
| Diabetes | 18 (11.5) | 5 (12.5) | > 0.99 |
| Maximal FiO2 (%) | 60 [39, 75] | 66 [50, 75] | 0.553 |
| **Other vasculitis manifestations** |  |  |  |
| Kidney | 139 (92.1) | 34 (91.9) | > 0.99 |
| Highest creatinine level (µM) | 289 [138, 498] | 468 [313, 670] | **0.004** |
| General signs | 130 (82.8) | 31 (79.5) | 0.802 |
| Skin | 24 (15.3) | 5 (12.5) | 0.846 |
| ENT | 42 (26.8) | 4 (10.3) | **0.049** |
| Eye | 10 (6.4) | 2 (5.3) | > 0.99 |
| Digestive tract | 12 (7.7) | 6 (15.4) | 0.24 |
| Peripheral nerve | 19 (12.1) | 3 (7.7) | 0.619 |
| Central nervous system | 3 (1.9) | 2 (5.1) | 0.567 |
| Heart | 10 (6.4) | 6 (15.0) | 0.144 |
| BVAS 2003 | 21 [19, 27] | 20 [18, 26] | 0.131 |
| SAPS II score | 38 [30, 48] | 54 [47, 64] | **<0.001** |
| Five factor score | 2 [2, 3] | 2 [2, 3] | **0.006** |
| **Treatments and outcomes** |  |  |  |
| PLEX | 122 (77.7) | 32 (80.0) | 0.921 |
| IV cyclophosphamide | 117 (74.5) | 28 (70.0) | 0.705 |
| Rituximab | 54 (34.4) | 11 (27.5) | 0.522 |
| Corticosteroid pulse | 150 (95.5) | 38 (95.0) | > 0.99 |
| Invasive mechanical ventilation | 74 (47.1) | 28 (70.0) | **0.016** |
| Neuromuscular blocking agents | 54 (34.4) | 20 (50.0) | 0.102 |
| Prone positioning | 33 (21.2) | 16 (40.0) | **0.024** |
| ECLS | 10 (6.4) | 6 (15.0) | 0.144 |
| Catecholamines | 53 (33.8) | 26 (65.0) | **0.001** |
| Acute RRT | 70 (44.9) | 32 (80.0) | **<0.001** |
| Alive on day 30 | 157 (100.0) | 12 (30.0) | **<0.001** |
| Hospital length of stay | 44 [34, 57] | 26 [24, 33] | 0.09 |
| ICU length of stay | 13 [7, 20] | 11 [7, 17] | 0.618 |
| Cause of death, any time |  |  | **0.002** |
| Cardiac arrest | 2 (8.7) | 1 (2.6) |  |
| Cancer | 3 (13.0) | 0 (0) |  |
| Cardiologic | 2 (8.7) | 0 (0) |  |
| Haemorrhage | 0 (0) | 5 (13.2) |  |
| Infectious | 12 (52.2) | 8 (21.1) |  |
| Neurological | 0 (0) | 2 (5.3) |  |
| Respiratory failure | 3 (13.0) | 6 (15.8) |  |
| Multiorgan failure | 0 (0) | 2 (5.3) |  |
| Vasculitis flare | 0 (0) | 12 (31.6) |  |

Results are N (%), or medians (interquartile range); FiO2: fraction of inspired oxygen; BAL: bronchoalveolar lavage; ENT: Ear-nose-throat; BVAS: Birmingham Vasculitis Activity Score; SAPS II: simplified acute physiology score; Bolded p-values are significant at the p<0.05 level.

### Supplementary Figure 1: Treatment regimen over time

### Supplementary figure 2: 30-day Kaplan-Meier survival curve according to clustering of 197 patients with AAV-vasculitis and severe intra-alveolar hemorrhage
